# Safe Food Coloring Agent as an Alternative to Eosin Stain

**DOI:** 10.7759/cureus.69817

**Published:** 2024-09-20

**Authors:** Samrangi Kar, Harish Kumar, Sangamesh NC, Silpiranjan Mishra, Atul Anand Bajoria, Mayurakshi Saha

**Affiliations:** 1 Department of Dentistry, Kalinga Institute of Dental Sciences, Bhubaneswar, IND; 2 Department of Oral and Maxillofacial Pathology, Kalinga Institute of Dental Sciences, Bhubaneswar, IND; 3 Department of Oral Medicine and Radiology, Kalinga Institute of Dental Sciences, Bhubaneswar, IND

**Keywords:** eosin stain, food colouring stain, haematoxylin and eosin stain, histopathology, organic

## Abstract

Introduction

Eosin stain is a commonly used histological dye that selectively binds to acidic structures in cells, imparting a color between pink and red. Eosin stain can be harmful due to its chemical composition. Inhaling eosin stain in powder form or as aerosolized droplets can cause irritation in the respiratory tract. To overcome the toxic effects of eosin, many naturally available organic substitutes have been tested in histopathology laboratories, including rose extract, beetroot stain, and curcumin. These natural stain alternatives provide effective staining of tissue while ensuring minimal risk to laboratory personnel.

Aim

The study was conducted to evaluate the efficacy of food coloring agents over eosin stain in histopathological investigations.

Materials and method

The study was carried out in Kalinga Institute of Dental Sciences, Kalinga Institute of Industrial Technology (KIIT) (Deemed to be University), Bhubaneswar, India. The sample size comprised 30 oral mucosal lesion blocks. After the preparation of four micron thick sections from each block, one was stained with hematoxylin and eosin (H&E) stain and the other with a food coloring agent. Sections were kept in food color stain for three minutes. Next, the sections were dehydrated, cleared, and mounted in dibutylphthalate polystyrene xylene (DPX). To evaluate the staining with H&E and food color, each section was scored 0 for inadequate staining, one for adequate, and two for excellent staining. The tissue components stained for the study were red blood cells (RBCs), collagen fibers, muscle fibers, epithelium overall, basement membrane, cell membrane, desmosomes, keratin, cytoplasm, and nuclei. Kolmogorov-Smirnov tests were used to calculate the inferential statistics for the different variables between the groups. The distribution of the study sample was found to be not normal; therefore, a nonparametric test of significance was applied.

Results

All the tissue components showed excellent staining by H&E (score 2). Among the tissue components, in most of the samples, keratin, cytoplasm, and RBCs showed excellent staining (score 2) on par with the H&E stain. Other tissue components showed no staining (score 0) to adequate staining (score 1). The basement membrane and cell membrane staining were not adequate (score 0). The nuclear staining by hematoxylin was unaffected by food color and was on par with normal H&E staining.

Conclusion

We conclude that tomato red food color, which is non-toxic, safe for the health of laboratory personnel, easy to dispose of, and environmentally friendly, could be used as a replacement for eosin in the routine H&E techniques. Our observations could be strengthened by increasing the sample sizes and modifying the stain preparation to ensure positive staining of all tissue components, thereby enhancing the results.

## Introduction

Medical laboratories conduct multitudes of investigative analyses on patient specimens. During these procedures, laboratory personnel employs a variety of potentially harmful chemicals, including radiochemicals, toxic chemicals, and pathogenic microorganisms [1].

Eosin stain is a commonly used histological dye that selectively binds to acidic structures in cells, imparting a color between pink and red. Eosin stain is frequently paired with hematoxylin, namely, in hematoxylin and eosin (H&E) staining, providing contrast to the blue-purple nuclei stained by hematoxylin. Eosin primarily stains collagen fibers, muscle fibers, cytoplasmic components, and red blood cells (RBCs), thereby aiding in the visualization of tissue architecture [2,3].

Eosin stain can be harmful due to its chemical composition. While it is generally safe when handled properly, prolonged exposure or inadvertent ingestion can lead to health risks. Eosin stain contains chemical compounds, such as eosin Y (eosin yellowish), which can be toxic if ingested or inhaled in large amounts. Direct contact with eosin stain can cause irritation to the skin, eyes, and mucous membranes. Prolonged exposure or contact with concentrated solutions of eosin stain may lead to redness, itching, and inflammation. Inhaling eosin stain in powder form or as aerosolized droplets can cause irritation in the respiratory tract, which can lead to breathing difficulties, especially in individuals with pre-existing respiratory conditions. Some individuals may be allergic to components of eosin stain, leading to reactions upon exposure. While eosin stain is biodegradable, large quantities discharged into water bodies can disrupt aquatic ecosystems [4].

In order to find an alternative to eosin, many naturally available organic substitutes have been tested in histopathology laboratories, such as rose extract, beetroot stain, curcumin, and food colors. These natural stain alternatives provide effective staining of tissue components while ensuring minimal risk to laboratory personnel and the environment [4-11].

Food coloring is a substance that is used to add color to food and beverages. Food coloring is non-toxic, organic, and environmentally friendly, and its ingredients are regulated by government authorities. Food coloring has been used in various non-food applications, including cosmetics, pharmaceuticals, crafting supplies, and medical devices. Furthermore, food coloring has been tested with good results in pathology laboratories by many authors as a replacement for eosin in routine H&E techniques [2]. We compared the staining ability of tomato red food color with the gold standard H&E technique in different tissues, including collagen fibers, muscle fibers, RBCs, and basement membrane, in oral mucosal tissue sections and analyzed whether it can be used in routine histopathology.

## Materials and methods

Food color stain

**Figure 1:**
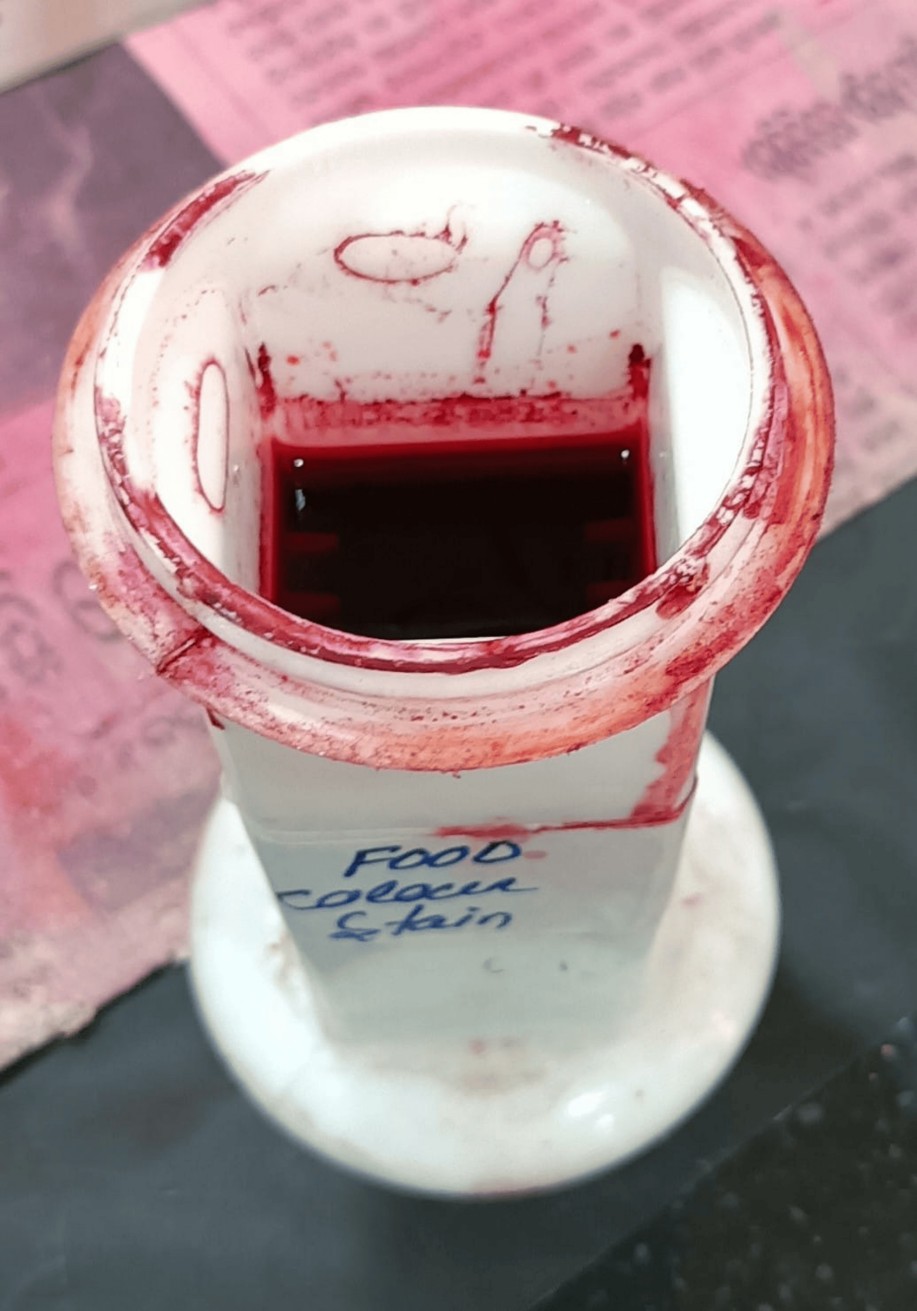
Prepared tomato red food color solution

Tomato red food color containing salt, synthetic food color (E122), and dye content of 32% (E110) was obtained from a commercial website; 1 g of powder was dissolved in 5 mL of 70% alcohol, stored for two days, filtered using filter paper, and used as stain (Figure 1) [2].

Sample

Thirty oral mucosal lesion blocks were selected from archives. Four micron thick sections were obtained on two separate slides from each block: one for the H&E stain group and the other for the food color stain group.

Procedure

The stained slide preparation involved several sequential steps. The sections were brought to water. Hematoxylin stain was applied for five to seven minutes, followed by a 35-minute tap water wash of the sections. An acid wash was performed by briefly immersing the slide in a solution of 2 mL of concentrated HCl in 100 mL of 70% alcohol, after which another tap water wash was performed. To achieve the blueing of the sections, the slide was dipped in saturated lithium carbonate solution. After a 23-minute running tap water wash, the slide was placed in the prepared food coloring stain for three minutes. Subsequently, the slides underwent consecutive dips, each two to four times, in three separate containers of 100% alcohol. Finally, the sections were cleared in xylene and mounted with dibutylphthalate polystyrene xylene (DPX), thereby completing the staining process.

Scoring

Each section was scored for both H&E and food color staining: 0 for inadequate, one for adequate, and two for excellent [5].

Statistics

Descriptive statistics were presented as frequency and percentage. Kolmogorov-Smirnov tests were used to calculate the inferential statistics for the different variables between the groups. The statistical constant was set at p < 0.05. Given the distribution of the study sample was not normal, a non-parametric test of significance was applied.

## Results

**Figure 2:**
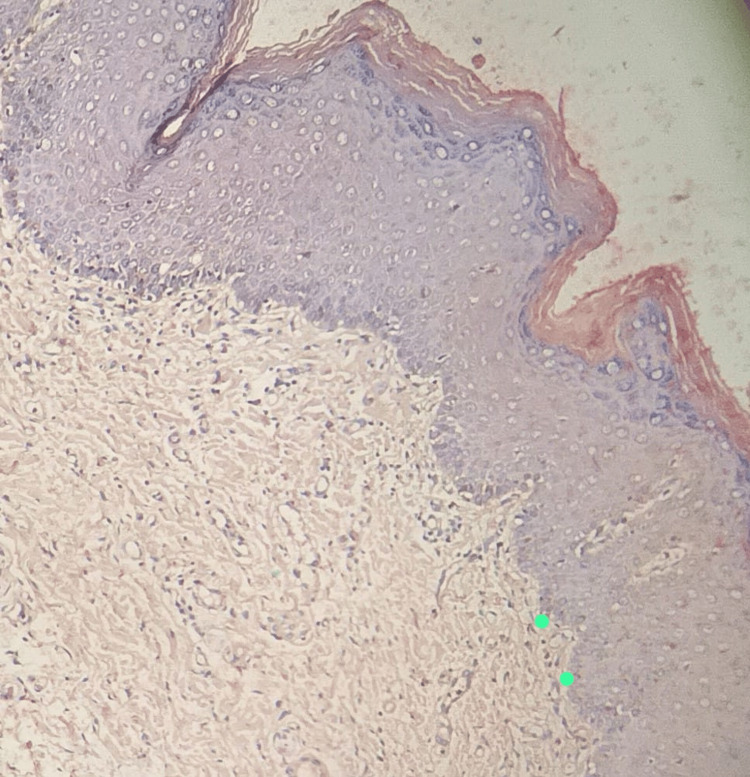
Photomicrograph showing excellent staining of keratin and inadequate staining of cell membranes and basement membrane (food color, 10×)

**Figure 3:**
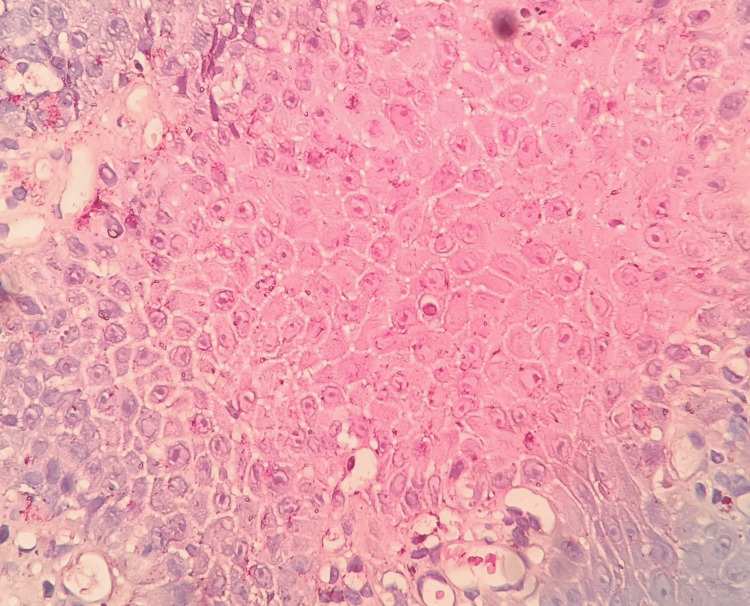
Photomicrograph showing adequate staining of desmosomes, nuclei, and cytoplasm (food color, 10×)

**Figure 4:**
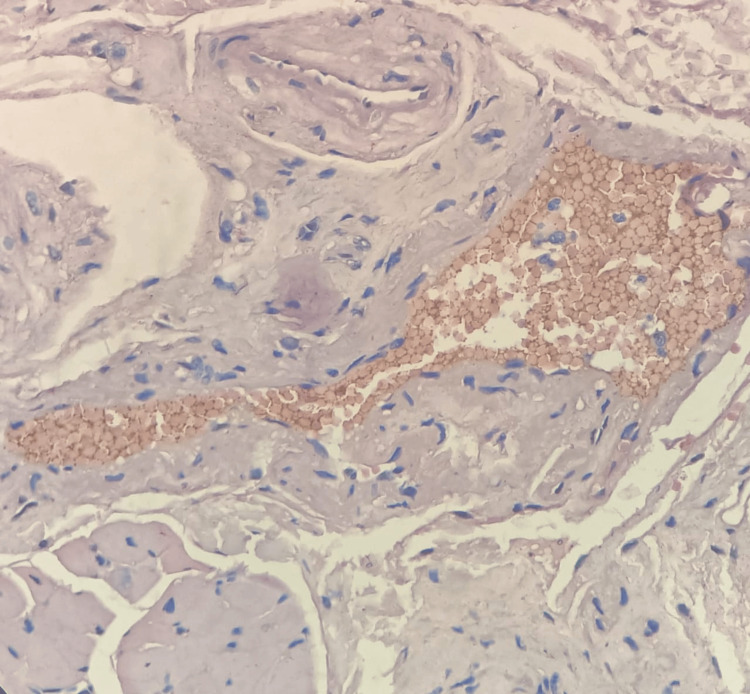
Photomicrograph showing excellent staining of red blood cells (food color, 10×)

All 30 test samples were positive for food color. Among the tissue components, keratin (Figure 2), cytoplasm (Figure 3), and RBCs (Figure 4) showed excellent staining (score 2) on par with the H&E stain in most of the samples (Table 1).

**Table 1:** Comparison of the parameters based on two different staining methods *Statistically significant

Parameters	H&E, frequency (n)				Food color, frequency (n)				Kappa score	z-score/p-value
	Total	0	1	2	Total	0	1	2		
Red blood cells	30	0	0	30	30	0	7	23	0.00	0.904/0.388
Collagen fibers	30	0	0	30	30	0	30	0	0.00	3.873/<0.0001*
Muscle fibers	30	0	0	30	30	0	30	0	0.00	3.873/<0.0001*
Epithelium overall	30	0	0	30	30	0	21	9	0.00	3.873/<0.0001*
Basement membrane	30	0	0	30	30	30	0	0	0.00	3.873/<0.0001*
Cell membrane	30	0	0	30	30	30	0	0	0.00	3.873/<0.0001*
Desmosomes	30	0	0	30	30	5	25	0	0.00	3.873/<0.0001*
Keratin	30	0	0	30	30	0	0	30	1.00	1.00
Cytoplasm	30	0	0	30	30	5	25	0	0.00	0.662/0.773
Nuclei	30	0	0	30	30	0	8	22	0.00	1.033/0.236

**Figure 5:**
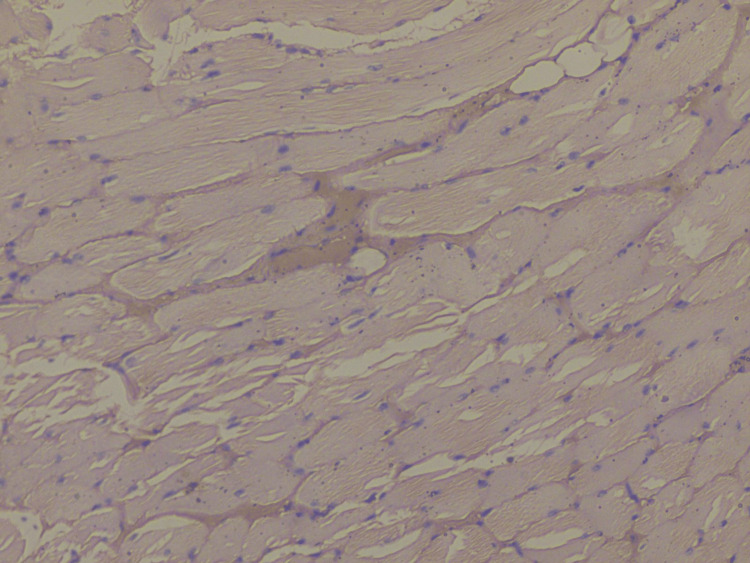
Photomicrograph showing adequate staining of muscle fibers (food color, 10×)

**Figure 6:**
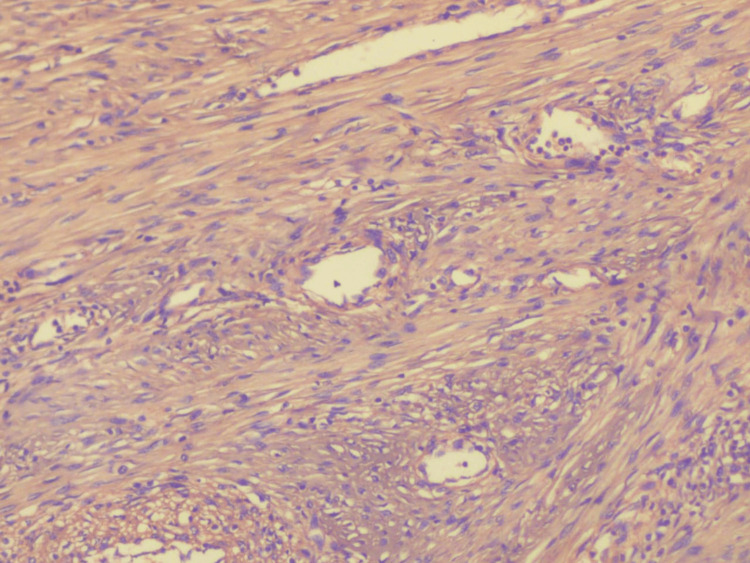
Photomicrograph showing adequate staining of collagen fibers (food color, 10×)

No two stains had a statistically significant difference in scores. Other tissue components (Figure 5 and Figure 6) showed no staining (score 0) to adequate staining (score 1), whereby the differences were statistically significant. The basement membrane (Figure 2) and cell membrane (Figure 3) stainings were not adequate (score 0). The nuclear staining by hematoxylin (Figure 6) was unaffected by food color and on par with normal H&E staining. The stability of the food color stain was intact after five months and will be followed up for five years.

## Discussion

The search for safe, non-toxic, organic alternatives for eosin in histopathology laboratories continues. Food colors approved as safe for humans have been tested by staining cytological smears and histopathological sections to ensure they are environmentally sound, safe for laboratory personnel, and can provide adequate diagnosis on par with the gold standard H&E technique [3].

We used tomato red food color as a replacement for eosin in routine H&E procedures and compared its staining ability for assessing different tissue components such as collagen, muscle, basement membrane, cell membrane, cytoplasm, keratin, and desmosomes in oral mucosal sections.

The nuclear staining by hematoxylin was not disturbed by the food color and was on par with the normal H&E technique. Therefore, both stains can be used synergistically.

We observed excellent staining of keratin, cell cytoplasm, and RBCs, which demonstrates their morphology and integrity. Adequate staining was seen in other tissue components. However, the basement membrane and cell membranes were negative for food color stain. Our findings are similar to those of previous studies that used eosin alternatives such as tomato red and green food colors, curcumin, saffron, beetroot, vegetable stains, and ginger [4-10].

Curcumin, extracted from turmeric, is a fluorescent biological stain widely used to highlight cellular components. Curcumin stains cytoplasm, keratin, and cell membranes, thereby revealing their structures under microscopy. Additionally, curcumin aids in visualizing RBCs and basement membranes, providing insights into their integrity and composition. Curcumin penetrates cells to stain nuclei, which facilitates studies on nuclear morphology and cell cycles [3,6-8].

Saffron, prized for its color and medicinal properties, functions as a biological stain with distinct effects on cellular components. It highlights keratin in tissues, such as skin and hair, facilitates cytoplasm visualization, and reveals RBC morphology in hematology. Additionally, saffron staining aids in studying the basement membrane's structure, cell membrane integrity, and nuclear morphology. This versatility makes saffron staining invaluable for detailed cellular analysis across various biological research applications [9,10].

Our tomato red stain did not adequately stain the basement membrane and cell membrane, whereas saffron and curcumin did. However, the preparation of our tomato red stain is easier and cheaper.

Beetroot, rich in betalain pigments, serves as a biological stain that effectively highlights cellular components such as cytoplasm and RBCs under microscopy. Beetroot provides a distinct red coloration useful for visualizing cellular structure and RBC morphology in hematology studies. In addition, beetroot can stain the basement membrane, thereby offering insights into the membrane structure. However, beetroot may not be as specific for highlighting keratin or nuclei compared to other specialized stains used in biological research. Overall, beetroot stain is valuable for its staining capabilities in cytoplasm and RBCs, which leverages its betalain pigments for cellular analysis. Our tomato red stain showed excellent staining of keratin and adequate staining of cytoplasm [12-16].

Vegetable stains, derived from various plants, can stain cytoplasm and potentially RBCs in microscopy. However, they may not be as effective for highlighting keratin, basement membranes, cell membranes, or nuclei compared to other specialized stains. Our tomato red stain showed excellent staining of keratin [4,17,18].

## Conclusions

We conclude that tomato red food color stain reveals the shape and integrity of RBCs, highlights keratin, and provides good visualization of cytoplasm. Tomato red food color stain can adequately reveal other tissue components, except the basement membrane and cell membrane. In addition, it is non-toxic, safe for the health of laboratory personnel, easy to dispose of, environmentally friendly, and it could be used as a replacement for eosin in routine H&E techniques. Our observations could be strengthened by increasing the sample sizes and modifying the stain preparation to ensure positive staining of all tissue components, thereby enhancing the results.
